# Method Development for Selected Bisphenols Analysis in Sweetened Condensed Milk from a Can and Breast Milk Samples by HPLC–DAD and HPLC-QqQ-MS: Comparison of Sorbents (Z-SEP, Z-SEP Plus, PSA, C18, Chitin and EMR-Lipid) for Clean-Up of QuEChERS Extract

**DOI:** 10.3390/molecules24112093

**Published:** 2019-06-01

**Authors:** Tomasz Tuzimski, Szymon Szubartowski

**Affiliations:** Departament of Physical Chemistry, Chair of Chemistry, Medical University of Lublin, 4A Chodzki Street, 20-093 Lublin, Poland; szymon.szubartowski95@gmail.com

**Keywords:** bisphenols, QuEChERS, dispersive solid phase extraction (d-SPE), zirconium dioxide-based sorbent, breast milk samples, milk from a can, HPLC-DAD, HPLC-QqQ-MS

## Abstract

*Background*: Identification and quantitative determination of analytes released from the packaging material is undoubtedly a difficult and tricky task, requiring the chemical analyst to develop an individual approach to obtain reliable analytical information. Unfortunately, it is still challenging for scientists to determine bisphenols at trace or even ultra-trace levels in samples characterized by a very complex, and often variable, matrix composition. *Objective*: Optimization and application of QuEChERS/d-SPE coupled with HPLC-DAD (and LC-QqQ-MS) method for the simultaneous determination of bisphenols (A, S, F, B, BADGE and derivatives) in milk samples from a can and breast milk samples have been performed. *Methods:* Concerning the analysis of unconjugated analytes, after the thawing and shaking the sample (5 mL breast milk or 10 mL milk samples from a can), it was transferred into a 50 mL polypropylene centrifuge tube. For the analysis of the total amount of analytes, prior to the extraction with acetonitrile, a deconjugation step was implemented in a tube by adding to sample, the an Isotopically Labelled Internal Standard (IS) solution (50 ng/mL) and 1 mL of the enzymatic solution with the β-Glucuronidase (3500 U/mL). The mix was homogenized and incubated for 16–18 h at 37 °C. Next, 10 mL of acetonitrile, and a QuEChERS salt packet (4 g anhydrous MgSO_4_, 1 g NaCl) were added. After shaking and centrifugation, the total acetonitrile layer was isolated in a polypropylene tube evaporate to dryness, and reconstitute in 1.2 mL acetonitrile. During d-SPE step the extract was transferred into a 15 mL polypropylene tube with Z-Sep and primary secondary amine (PSA). Next, shake the tube, store in fridge, and centrifuge for 15 min. The acetonitrile supernatant was obtained with a pipette and evaporated to dryness. Mixture MeOH: water (20:80, *v*/*v*) were added to the dry residue and the extract was reconstitute in 200 μL and analyzed by HPLC-DAD and HPLC–QqQ-MS equipment. *Conclusion*: Six different salts during d-SPE step were evaluated such as: zirconium dioxide-based sorbent (Z-Sep, Z-Sep Plus), primary secondary amine (PSA), octadecyl (C18), EMR-Lipid, Chitin and also their mixtures. Negligible matrix interference was observed for most of the analytes due to application of Z-Sep and PSA in dispersive-solid phase extraction clean-up step. Extraction of target analytes was performed using QuEChERS/d-SPE cleanup, and presents good performance for selected analytes with recoveries in the range of 15–103% and relative standard deviations (RSD) less than 10% in breast milk samples.

## 1. Introduction

Bisphenol A (BPA) is a high-production volume industrial chemical mainly used as a monomer in the production of polycarbonate plastics (~80%) and epoxy resins (~18%) [1,2]. Both of these polymers are widely used as food contact materials (namely, polycarbonate plastics in reusable food and drink containers, in tableware, and in water pipes, and epoxy resins as inner coatings of cans and lids of glass jars and bottles for food and beverages). In the last years endocrine disrupting compounds (EDCs) have become the chemical group of special concern due to their ability to interfere with hormonal system. Bisphenol A (BPA) has attracted high concern because of its endocrine-disrupting effects and its widespread occurrence. As indicated in numerous research, bisphenols may be washed off the material surface and transferred to food or individual elements of the environment due to the interaction with food ingredients or the influence of external factors. Moreover, many of these xenobiotics are characterized by lipophilicity; therefore, they are able to easily pass through biological membranes and penetrate living cells, and thus be subject to bioaccumulation in various kinds of tissue and organs. 

Since 2006, the European Food Safety Authority (EFSA) has conducted several scientific assessments on BPA. The tolerable daily intake (TDI) was decreased from 50 μg/kg bw/day to a temporary TDI of 4 μg/kg bw/day by the European Food Safety Authority (EFSA) in 2015 owing to new data and refined methodologies [3,4,5]. The restrictions have led to the BPA structural analogs, such as bisphenol S (BPS), bisphenol F (BPF), bisphenol B (BPB), bisphenol C (BPC), bisphenol E (BPE), bisphenol M (BPM), bisphenol P (BPP), bisphenol Z (BPZ), bisphenol AF (BPAF), bisphenol AP (BPAP), bisphenol BP (BPBP), bisphenol FL (BPFL), dihydroxydiphenyl ether (DHDPE), gradually entering the market. However, Bisphenol S (BPS) and bisphenol F (BPF) are nowadays the most commonly used BPA substitutes, predominantly in the manufacturing of epoxy resins, polyesters and polycarbonate plastics. 2-[[4-[2-[4-(oxiran-2-ylmethoxy)phenyl]propan-2yl]phenoxy] methyl]oxirane (BADGE) and related compounds, e.g., 3-[4-[2-[4-(oxiran-2-ylmethoxy)phenyl]propan-2-yl]phenoxy]propane-1,2-diol (BADGE·H_2_O), 3-[4-[2-[4-(2,3-dihydroxypropoxy)phenyl]propan-2-yl]phenoxy]propane-1,2-diol (BADGE·2H_2_O), 1-chloro-3-[4-[2-[4-(3-chloro-2-hydroxypropoxy)phenyl] propan-2-yl]phenoxy]propan-2-ol (BADGE·2HCl) are used mainly for the production of the inner coatings of packaging materials. Considering that other compounds are suspected of showing similar or even higher endocrine and toxic effects than BADGE, it seems necessary to update the existing legal regulations. To ensure maximum consumer safety, the European Commission, by virtue of the Commission Regulation no. 10/2011, has established Specific Migration Limits (SML) for individual compounds. The EU specified a maximum SML of 9 mg/kg for foodstuff or simulated liquid for the sum of BADGE and its hydrolytic products and 1 mg/kg for chlorinated derivatives [6]. A new European Regulation (EU, 2018) has tightened the restrictions on the use of BPA in food contact materials. This Regulation has lowered the migration limits (0.05 mg of BPA per kg of food), expands the ban of use of BPA in baby bottles and prohibits the migration of BPA from varnishes or coatings applied to materials in contact with food for infants and children 0-3 years old [7]. 

Common steps in sample treatment for most of the analytical methods reported for mixtures of bisphenols and derivatives include sample pretreatment, extraction of analytes from the matrix, cleanup of the extracts to remove interferences, and concentration to achieve the desired sensitivity. Analyte separation and quantification has been almost exclusively carried out by LC-MS/MS or GC-MS, in the last case prior derivatization, but LC-fluorescence detection (LC-FLD) has also found some applications [2]. 

Incontestable progress was made during the past years regarding the development of the techniques of preparation of samples for analysis. Various methods were used to extract food contact materials contaminants from foodstuffs and packaging such as Solid Phase Extraction (SPE), Solid Phase Microextraction (SPME), Stir Bar Sorptive Extraction (SBSE), Hallow-Fiber Liquid Phase Microextraction (HFLPME), QuECHERS (Quick, Easy, Cheap, Effective, Rugged, and Safe), Dispersive Liquid-Liquid Microextraction (DLLME) or Focused Ultrasonic Solid-Liquid Extraction (FUSLE) have been successfully applied [2,6,8]. 

The choice of an appropriate analytical method for separation and final determination is closely related to the properties of the target compounds. Various analytical procedures have been reported for the determination of bisphenols in different biological samples. Generally, in the case of determination of BPA and related compounds where a clear preference cannot be determined, both LC and GC are used. LC-MS/MS has become the first choice for separation and quantitation of mixtures of bisphenols, diglycidyl ethers and chlorinated derivatives [2]. GC-MS continues as a valuable alternative for determination of mixtures of bisphenols. BPA urinary concentrations were measured using GC-MS/MS in 315 men under 45 years of age with normal sperm concentration (≥15 mln/mL) recruited from a male reproductive health clinic [9]. BPA was detected in 98.10% of urine samples, with a median concentration of 1.87 µg/L (1.63 µg/g creatinine). A multiple linear regression analysis identified a positive association between the urinary concentrations of bisphenol A 25th–50th percentile and total sperm sex chromosome disomy (*p* = 0.004) [9]. 

LC-MS/MS is generally preferred to analyze these compounds, because generic and fast methods for screening purposes can be developed, and the derivatization steps needed for a GC-MS analysis can be omitted [10]. A more simple technique, LC-FLD, has found interesting applications related to the determination of mixtures of bisphenols and/or diglycidyl ethers in food, environmental, and biological samples [2,6,8]. 

Breast milk is one possible route of exposure to environmental chemicals, including bisphenols for breast-fed infants. Breast milk is the major or exclusive energy source for infants up to 6 months of age, as well as a reflection of internal exposure level of contaminants in mothers and fetuses. Therefore, breast milk is frequently monitored for exposure assessment of BPA. BPA analogs and halogenated derivatives have been reported as having similar or even greater toxic effects compared with those of BPA. However, there is little information on the occurrence of BPA-related compounds in breast milk. One reason is the lack of analytical methods for the simultaneous determination of bisphenols. The reported methods mainly focused on the detection of BPA in breast milk. Since the human milk provides a dosimeter of prenatal exposure, it is considered as a biomarker of previous bisphenol maternal exposure and a target biological matrix for priority exposure assessment. 

The on-line SPE-HPLC-MS/MS was applied for determination of bisphenol A in human milk [11,12]. Niu et al. analyzed bisphenols in breast milk samples by UPLC-MS/MS on C18 column with mixture of acetonitrile, water, and formic acid [13]. C18 column and mobile phase containing acetonitrile and water were applied for LC-MS/MS determination of bisphenol A in breast milk samples [14]. In the procedure liquid-liquid extraction was used for sample preparation before chromatography analysis. 

In the literature, there are not many reports on application of the QuEChERS approach for the analysis of bisphenols in breast milk samples. Originally, QuEChERS was introduced for pesticides residue analysis in high moisture fruits and vegetables, but more recently it is gaining significant popularity in the analysis of a broad spectrum of analytes in huge variety of samples [15,16,17]. This technique involves liquid-liquid partitioning using acetonitrile and purifying the extract using dispersive solid-phase extraction (d-SPE). The QuEChERS technique has important advantages over most traditional extraction methods. One of the relevant pros is its flexibility, because due to the possibility of introduction of different d-SPE sorbents it may be useful for analysis of broad spectrum of analytes in various sample types with different properties [17]. 

There is a great concern about exposure of human fetuses, neonates, and infants to bisphenols because of the sensitivity of the developing organs and brain to endocrine disrupting chemicals. Considering that bisphenols can cross the placental barrier, the fetus remains exposed to these compounds. The evaluation of “in utero exposure” to bisphenols, through the analysis of biological fluids from pregnant or nursing mother (i.e., blood, urine, breast milk, colostrum), the fetus or newborn infant (i.e., meconium, umbilical cord blood, neonatal urine), and from both the fetus and the mother (i.e., placental tissues, amniotic fluid), would allow for a better understanding and a more concrete picture into the exposure of the most vulnerable segment of the human population. 

The aim of previous study [18] was to optimize QuEChERS-based sample preparation procedure for selected bisphenols analysis in breast milk samples by high performance liquid chromatography coupled with modern detection techniques (HPLC-DAD and LC-QqQ-MS). In previous experiments [18] during dispersive solid-phase extraction (d-SPE) step were applied zirconium based sorbents (50 mg Z-Sep or 45 mg Z-Sep and 5 mg Z-Sep Plus). Moreover, some samples were prepared with the addition (1%) of acetic or (1%) formic acids [18]. 

In the literature, there are no too many reports on the application of the QuEChERS approach for the analysis of bisphenols in milk. However, to our knowledge, only Niu et al. [13], Tuzimski et al. [18], and Deceuninck et al. [19] have studied human milk levels of BPF and/or BPS in small populations (n = 20–30 samples). In another paper, the study addresses the presence of bisphenols A (BPA) and its analogs bisphenol F (BPF) and S (BPS) in milk of 120 mothers living in Valencia (Spain) and participating in the BETTERMILK project (in the year 2015) was described by Dualde et al. [20]. 

The aim of this study was to optimize QuEChERS-based sample preparation procedure for selected bisphenols (A, S, B, F, BADGE and derivatives) analysis in breast milk samples. The sample preparation conditions were further more optimized. Due to the of lack of scientific data concerning analytical methods for the determination of a selected of BPA analogues, the aim of this research was to develop an easy, fast, highly sensitive, and robust method for human biomonitoring of these chemicals. The aim of this study was to determine the occurrence of BPA, BPF, BPB, BPS, BADGE and their derivatives in breast milk samples and study the factors influencing the bisphenols levels. Different six sorbents and their mixtures were evaluated for the removal of matrix interferences (MIs) and the recovery of analytes. In this study authors developed an easy and cost-effective HPLC-DAD method for analysis of bisphenols. The procedures were preliminary validated and applied to analysis of different milk samples, such as milk from a can and breast milk samples. 

## 2. Results and Discussion

### 2.1. Sample Preparations

**Figure 1:**
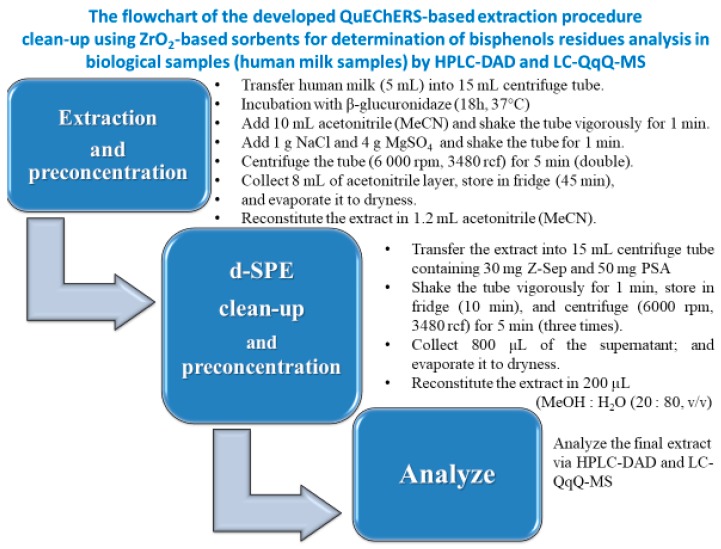
The flowchart of developed QuEChER-based extraction procedure clean-up using ZrO_2_-based sorbents for determination of bisphenols residues analysis in biological samples (human milk samples) by HPLC-DAD and LC-QqQ-MS.

The flowchart of the procedure is presented (Figure 1). The introduction of pre-concentration steps before chromatographic analysis led to increase in sensitivity of whole analytical procedure. During the elaborated procedure 25-fold analytes pre-concentration is achieved (Figure 1). Described extraction procedure was assessed in terms of recovery, repeatability and matrix effect; furthermore, method limits of quantification (mLOQs) were established. 

### 2.2. Chromatographic Conditions

**Figure 2:**
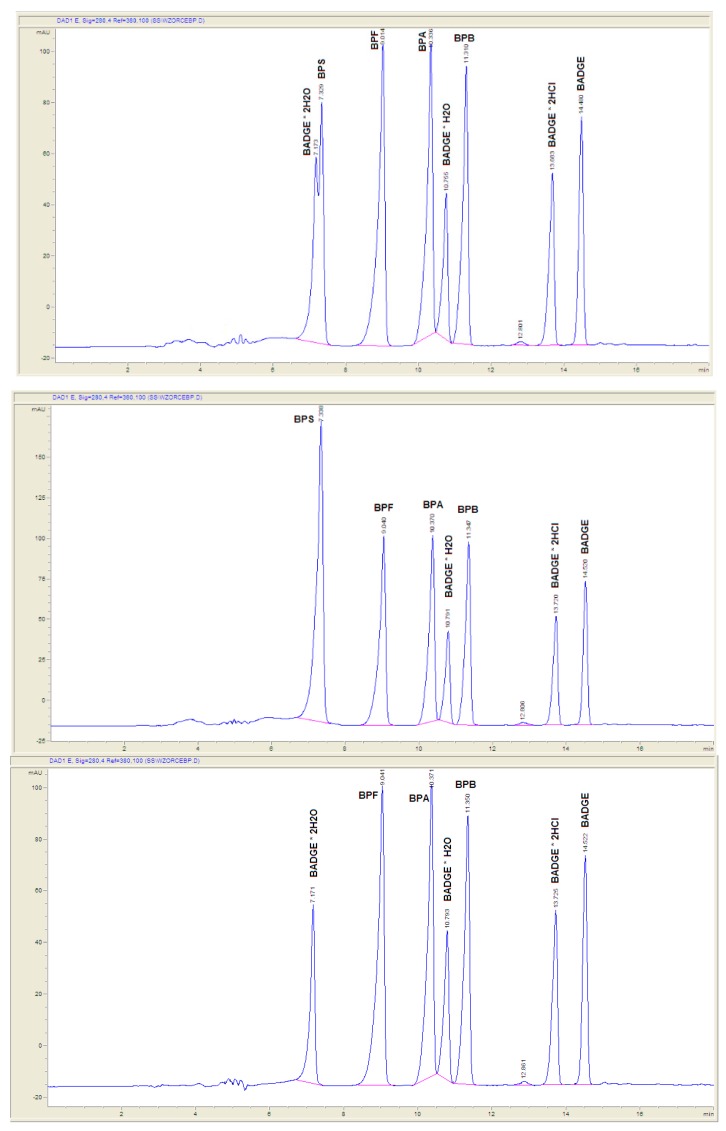
Chromatograms showing separation of standards of eights bisphenols (top—all eights standards; middle—without BADGE·2H_2_O; bottom—without BPS) by HPLC-DAD with application of following chromatographic conditions: the stationary phase Scherzo SM-C18; the mobile phase consisted of 50 mM HCOOH in water (component A, pH = 2.55) and 50 mM HCOOH in MeCN (component B). Gradient elution at 0.4 mL min^−1^ mobile phase flow was applied. The gradient program was as follows: 0–10 min from 40% eluent B to 100% B; 10–16 min isocratic 100% B.

Chromatographic conditions utilized in the experiments were preliminary elaborated by the authors previously [18]. Chromatographic separation using Scherzo SM-C18 column provided satisfactory results for a wide range of bisphenols. The applied gradient elution program allowed appropriate separation of the analytes under investigation in a single chromatographic run, as presented in Figure 2 and Table 1. Gradient elution at 0.4 mL min^−1^ mobile phase flow was applied. The gradient program was as follows: 0-10 min from 40% eluent B to 100% B; 10–16 min isocratic 100% B (Figure 2). 

HPLC–DAD applied in the experiments allows determination and quantitation of the analytes at ng per mL level of the milk samples. Instrumental limits of detection and quantitation (LODs and LOQs) were from 53 to 306 ng mL^−1^ and from 159 to 926 ng mL^−1^; and from 142 to 693 ng mL^−1^ and from 430 to 2102 ng mL^−1^ for unconjugated and conjugated analytes with β-glucuronidase (Table 1), respectively. 

### 2.3. Optimization of QuEChERS-Based Procedure

Undoubtedly, sample preparation is crucial for high-throughput multiresidue methods (MRMs), especially if UV–VIS or DAD detection is applied. The lower selectivity of HPLC-DAD in comparison with LC-MS and/or LC-MS/MS should be overcome by appropriate sample purification to eliminate as many interfering compounds as possible. If it succeeds, HPLC-DAD could be less expensive alternative to methods relaying on MS detection. 

In our study, we applied QuEChERS approach for bisphenols residue extraction from milk samples from a can. Sample amount, solvent type and volume, salts type and amount, buffer additives, extract volume for clean-up and d-SPE sorbent type and amount should be adjusted to obtain satisfactory extraction efficiency and sample purification. Milk samples from a can, considered as moderately fatty (6% fat content), need some special effort focusing on extraction and extract clean-up. It may be performed by application of different sorbents in d-SPE step of the QuEChERS procedure. Application of various sorbents in dispersive-solid phase extraction allows getting rid of undesirable matrix compounds. 

**Figure 3:**
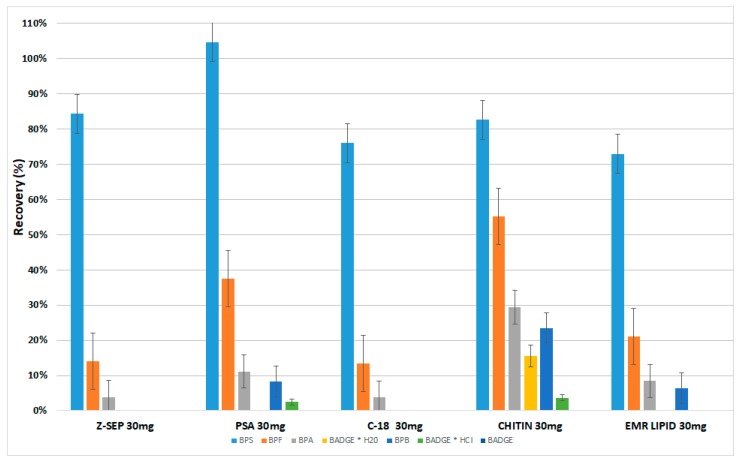
Influence of different single sorbents applied in dispersive solid-phase extraction (d-SPE) clean-up step on recovery of bisphenols in sweetened milk samples from a can analyzed by QuEChERS-HPLC-DAD.

In our preliminary experiments, we evaluated for this purpose various sorbents such as primary secondary amine (PSA), octadecyl (C18), and relatively new commercially available dispersive phases based on zirconium dioxide modified silica particles (Z-Sep) and both zirconia and C18 dual bonded on the same silica particles (Z-Sep Plus). Additionally, we tested EMRL-Lipid and Chitin which were recommended to remove lipids (Figure 3). After the application of Z-Sep Plus during d-SPE, the values of recoveries of all analytes were significantly lower in comparison to the recoveries values of analytes obtained during d-SPE experiments with Z-Sep (therefore these results are not shown in Figure 3). During this stage of experiments, due to the very low recovery values of the analytes, the Z-Sep Plus was excluded from continued research. 

**Figure 4:**
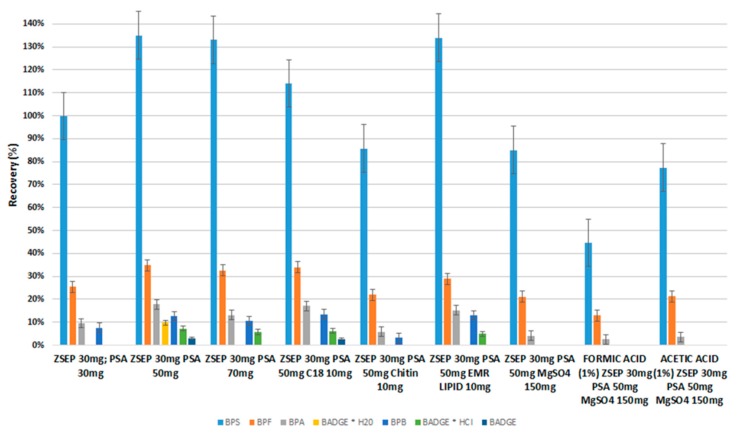
Influence of different combinations of sorbents applied in d-SPE clean-up step on recovery of bisphenols in sweetened milk samples from a can analyzed by QuEChERS-HPLC-DAD.

The extraction procedure must be optimized for particular analysis. During QuEChERS procedure, the single-packaged sorbents were used to prepare the sets (their mixture of sorbents/salts) applied during the dispersive-solid phase extraction clean-up step. Negligible matrix interference was observed for most of the analytes due to application of 50 mg PSA and 30 mg Z-Sep or 50 mg PSA, 30 mg Z-Sep and 150 mg MgSO_4_ sorbents (their mixture was prepared by used single-packaged sorbents) in d-SPE extraction clean-up step (Figure 4). In our opinion, the best analytical performance was achieved using combination of 50 mg PSA and 30 mg Z-Sep for milk extracts clean-up. The main function of PSA is removal of co-extracted constituents such as fatty acids, sugars, and ionic lipids, whereas Z-Sep and Z-Sep Plus are applied to adsorb majority of fatty non-polar interferences (e.g., lipids). 

We tried to choose the best set of salts during this stage, which is a compromise allowing for proper purification of the matrices from interferences, and which at the same time, will not significantly reduce the analytes recovery value. During d-SPE experiments, it was attempted to optimize this stage by using sets of salts such as 50 mg PSA and 30 mg Z-Sep or 50 mg PSA, 30 mg Z-Sep and 150 mg MgSO_4_ sorbents, which satisfactorily allows to purge the matrix and at the same time, the values of recoveries of selected bisphenols (e.g., S, F, A, and B) are still satisfactory. Bisphenol S is an analyte with the most polar properties, which has a sulfone group. Therefore, BPS has a lower affinity for the zirconia salts used during the d-SPE step, so that its recovery value is the greatest. In our opinion, the main reason of the low recoveries of certain bisphenols (e.g., F, A, and B) compared to the recovery value of bisphenol S, may result from stronger interactions of analytes with higher hydrophobic properties with Z-Sep salt during the d-SPE step. 

The introduction of pre-concentration steps before chromatographic analysis led to increase in sensitivity of whole analytical procedure. During the elaborated procedure 25-fold analytes pre-concentration is achieved (Figure 1). Described extraction procedure was assessed in terms of recovery, repeatability and matrix effect; furthermore, method limits of quantification (mLOQs) were established. 

### 2.4. Recovery Studies

**Figure 5:**
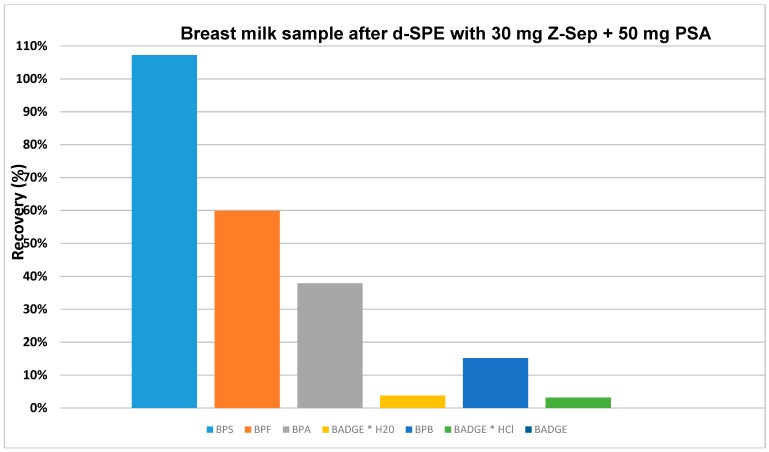
Influence of 30 mg Z-Sep and 50 mg PSA sorbents applied in d-SPE clean-up step on recovery of bisphenols in breast milk samples analyzed by QuEChERS-HPLC-DAD.

Recovery studies were carried out at three spiking levels of 500 ng mL^−1^ (HPLC-DAD), 50 and 5 ng mL^−1^ (LC-QqQ-MS). From the bisphenols under investigation, some of them showed satisfactory recoveries for samples, in which fortified level equals 500 ng mL^−1^ (HPLC-DAD). The recovery values were ranging between 15 and 135% with RSD% less than 18% (milk samples from a can); and as shown in Figure 5 between 15 and 107% with RSD% less than 10% (breast milk samples). 

For comparison, Dualde et al. applied a method relaying on dispersive solid-phase extraction followed by LC-MS/MS for the analysis of bisphenols in breast milk samples of 120 mothers living in Valencia (Spain) [20]. The BPA, BPS, and BPF that were selected by the authors for the experiments were the same as in this paper. However, the procedure proposed by Dualde et al. is slightly simpler and faster; application of d-SPE AOAC kit (salt/sorbents mixture consisting of 400 mg PSA, 400 mg C18 and 1200 mg MgSO_4_) is more expensive than 50 mg PSA and 30 mg Z-Sep (or 50 mg PSA, 30 mg Z-Sep and 150 mg MgSO_4_) utilized in our method. The same applies to much higher costs of instrumentalization. 

Average analyte recovery rates for spiked at 5 and 50 ng/mL samples for the QuEChERS procedures were an acceptable for selected bisphenols such as BPS, BPF, and BPA, 102%/95%, 68%/63%, and 39%/35% for two fortified levels 5 ng mL^−1^ and 50 ng mL^−1^, respectively. 

Application of the LC-MS/MS conditions described in the Experimental section allowed proper separation, identification, and quantification of the selected bisphenols. Sufficient sensitivity was achieved applying these conditions, with LOQs ranging from 0.10 to 0.25 ng mL^−1^ (Table 2). Recovery studies conducted at two spiking levels of 5 ng mL^−1^ and 50 ng mL^−1^ proved that the elaborated extraction procedures support the possibility of BPS, BPF, and BPA, and residue determination in breast milk samples. 

### 2.5. Application of the Procedure to the Analysis of Natural Samples

#### 2.5.1. Application of the Procedure to the Analysis of Sweetened Condensed Milk Samples from a Can

**Figure 6:**
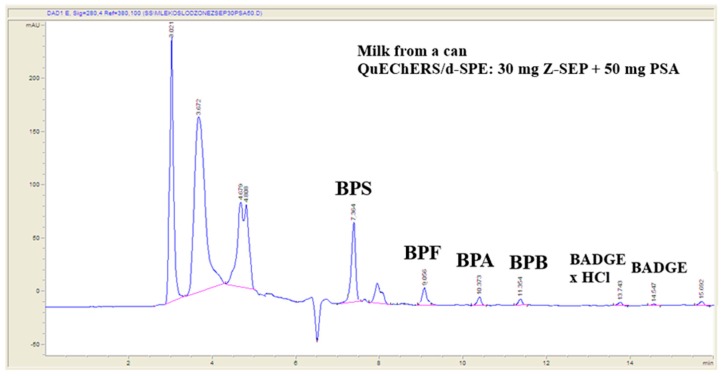
Example chromatogram of sweetened milk samples from a can showing detected bisphenols residues.

The validated method was applied to the analysis of bisphenols in milk samples from a can purchased in local market. Ten samples were analyzed utilizing extraction and chromatographic conditions described in experimental subsection of the paper. Successful purification of extracts utilizing PSA, Z-Sep and Z-Sep Plus, as well as presence of enrichment steps in the extraction procedure, allows determination of bisphenols at low ng mL^−1^ level. Residues of BPS (0.3–0.7 ng mL^−1^, n = 4), BPA (0.6–0.9 ng mL^−1^, n = 3), and BPF (0.4–0.6 ng mL^−1^, n = 2) were detected in sweetened milk samples from a can. Examples of chromatogram of natural sample showing detected bisphenols are presented in Figure 6. Detection and quantification of bisphenols residues in natural samples, even at lower level then validated mLOQs, confirms usefulness of the elaborated analytical procedure. 

#### 2.5.2. Application of the Procedure to the Analysis of Breast Milk Samples

**Figure 7:**
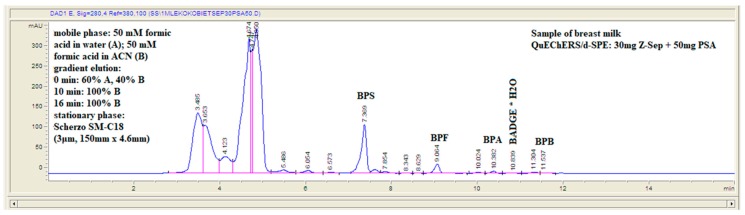
Example chromatogram of breast milk samples showing detected bisphenols residues.

**Figure 8:**
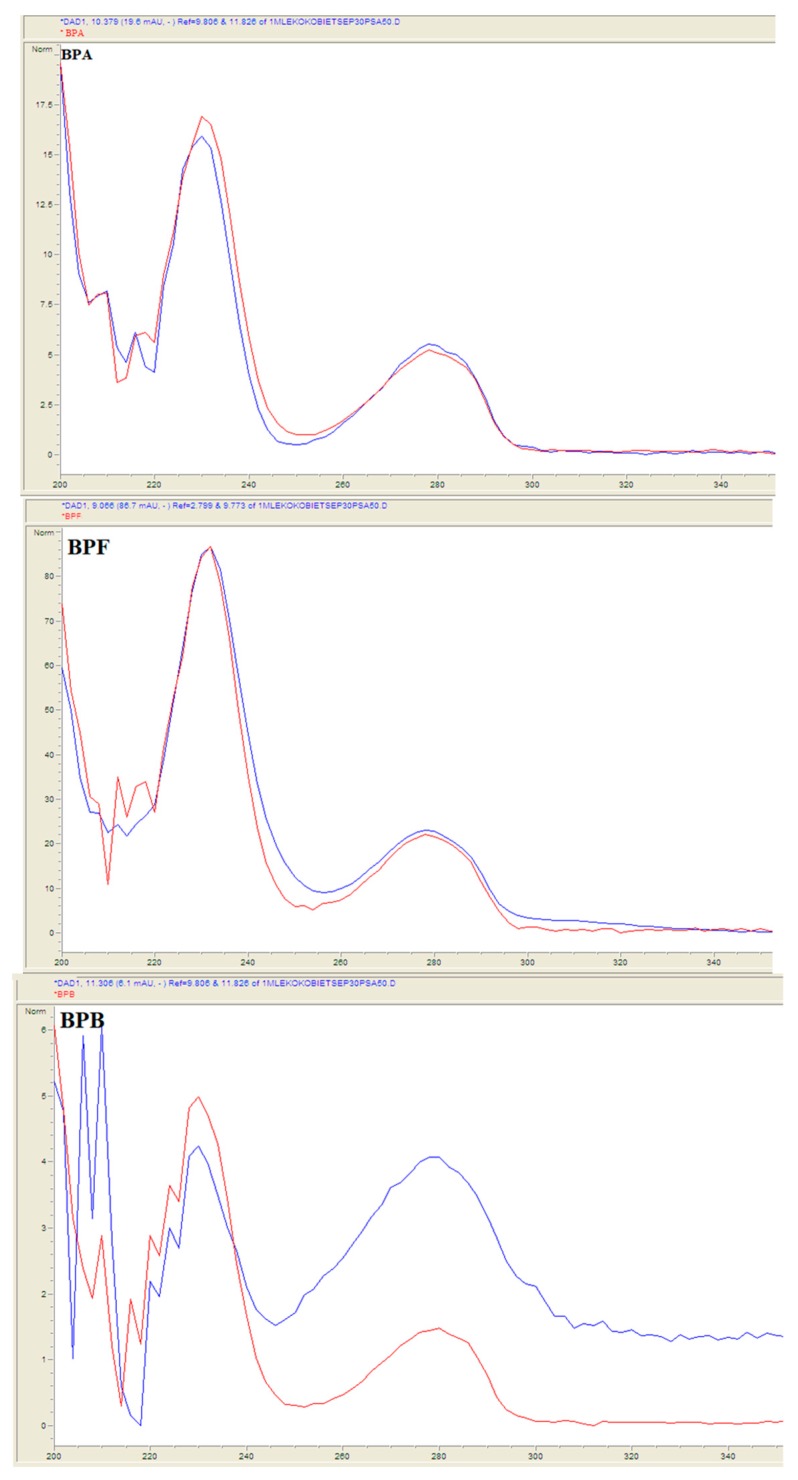
Examples correlations between UV spectra of analytes and library bisphenols standards in breast milk sample.

Breast milk samples were obtained from 50 voluntary donors: from 25 healthy voluntary-women donors in Lublin, Poland (urban area) and from 25 healthy voluntary-women donors Lubelskie voivodeship (rural area). Samples collection was conducted from June to August in 2018 and from March to April in 2019. Fifty samples were prepared utilizing QuEChERS-based extraction procedure and analyzed under chromatographic LC-QqQ-MS conditions described in the Experimental section. Residues of bisphenols BPS, BPF, BPA, BPB, and BADGE·H_2_O were detected in samples (Figure 7). Suitable matches between UV spectra of the residues identified in natural samples and library standards were observed (Figure 8). The slight differences in the spectra are due to the fact that the bisphenol standards were dissolved in methanol, and the samples concentrated in a mixture of methanol and water. Concentrations of identified bisphenols residues ranged from 0.21 to 0.69 ng mL^−1^ in breast milk samples (Table 3). 

## 3. Materials and Methods

### 3.1. Chemicals and Reagents

#### 3.1.1. Analyte Standards

Standards for the bisphenols under investigation, such as bisphenol A (BPA), S (BPS), bisphenol F (BPF), bisphenol B (BPB), 2-[[4-[2-[4-(oxiran-2-ylmethoxy)phenyl]propan-2yl]phenoxy] methyl]oxirane (BADGE), 3-[4-[2-[4-(oxiran-2-ylmethoxy)phenyl]propan-2-yl]phenoxy]propane-1,2-diol (BADGE·H_2_O), 3-[4-[2-[4-(2,3-dihydroxypropoxy)phenyl]propan-2-yl]phenoxy]propane-1,2-diol (BADGE·2H_2_O), 1-chloro-3-[4-[2-[4-(3-chloro-2-hydroxypropoxy)phenyl] propan-2-yl]phenoxy]propan-2-ol (BADGE·2HCl), and isotope labeled standard of bisphenol A (BPA-d_16_), were obtained from Sigma–Aldrich (Bellefonte, PA, USA). Isotope labeled standards of bisphenol F (BPF-d_10_) and S (BPS-d_8_) were purchased from Toronto Research Chemicals (Toronto, Canada) and CDN Isotopes (Quebec, Canada), respectively. The standard purity indicated by the manufacturers for all of the reference standards was ≥98.0%. 

#### 3.1.2. Solvents and Mobile-Phase Solutions

LC-MS grade acetonitrile (MeCN) and methanol (MeOH) were obtained from E. Merck (Darmstadt, Germany). LC-MS grade water were purchased from Sigma-Aldrich (St. Louis, MO, USA). Moreover, deionized water (0.07–0.09 µS cm^−1^) was obtained by means of Hydrolab System (Gdansk, Poland) in our laboratory. 

Solvents and reagents were also avoided to contact with plastic materials. Furthermore, the polypropylene material used for sample analysis and reagents (including QuEChERS) were checked previously for BPA contamination. All glassware was cleaned with methanol prior to the analysis. Moreover, quality control blanks were periodically prepared and analyzed. All solvents were checked for the presence of the target analytes before use. 

Individual stock standard solutions were prepared in methanol and were stored in screw capped glass tubes at −4 ± 2 °C in the dark. A bisphenols standards mixture containing all the analytes was prepared by combining suitable aliquots of each individual standard stock solution and diluting them methanol and was stored at 4 ± 2 °C for up to one month. This mixture was used for the calibration preparation, as well as for fortification of the breast milk samples. 

#### 3.1.3. Enzymatic Standard and Solutions

β-Glucuronidase from *Helix pomatia* type H1 was obtained from Sigma Aldrich (St. Louis, MO, USA). The enzymatic solution was prepared weekly by disolving the β-glucuronidase purified powder in ammonium acetate 1M (acetic acid was added to pH = 5) to obtain a solution of 3500 U/mL. 

#### 3.1.4. QuEChERS Salts and Sorbents

Anhydrous magnesium sulphate (MgSO_4_) and sodium chloride (NaCl) were obtained from POCH (Gliwice, Poland). Furthermore, single-packaged sorbents used to prepare the sets (their mixture) used during the d-SPE stage such as clean primary secondary amine (PSA), chitin and octadecyl (C18) were obtained from Sigma–Aldrich (Bellefonte, PA, USA). QuEChERS Enhanced Matrix Removal – Lipid (EMR-Lipid) was obtained from Agilent (Santa Clara, CA, USA). 

### 3.2. Breast Milk Sample Collection

Breast milk samples were obtained from 50 healthy voluntary-women donors in Lublin, Poland (urban) and Lubelskie voivodeship (rural). Samples collection was conducted from June to August in 2018 and from March to May in 2019. All the participants (urban (n = 25) and rural (n = 25)) provided a breast milk sample in 3 days (2019) and 2 weeks after birth (2018). After cleaning their breasts with abundant clean water, samples were collected by the mothers in a glass container using a BPA-free breast pump. All samples were collected in the glass bottles and immediately, on a regular basis analyzed or frozen immediately at −8°C until analysis. This study was approved by the ethics committee of the Medical University of Lublin, Poland (No. KE-0254/271/2018). 

### 3.3. Sample Preparation of Sweetened Condensed Milk from a Can and Breast Milk Samples

Concerning the analysis of unconjugated analytes, after the thawing and shaking the sample (5 mL breast milk or 10 mL milk samples from a can), it was transferred into a polypropylene centrifuge tube (50 mL, checked: free of BPA). 

For the analysis of total analytes, prior to the extraction with acetonitrile, a deconjugation step was implemented in a tube by adding 10 mL of sample, an Isotopically Labeled Internal Standard (IS) solution (500 ng/mL for sweetened milk samples from a can and for breast milk samples—both for HPLC-DAD; 5 ng/mL and 50 ng/mL for LC-QqQ-MS), and 1 mL of the enzymatic solution with the *β-*glucuronidase (3500 U/mL). The mix was homogenized and incubated for 17 h at 37 °C. 

Next, 10 mL of acetonitrile was added, which was shaken vigorously for 1 min. Then, a QuEChERS salt packet (4 g anhydrous MgSO_4_, 1 g NaCl) was added and shake the tube for 1 min. After shaking and centrifugation (6000 rpm, 3480 rcf, 10 min), the total acetonitrile layer (~8 mL) was isolated in a polypropylene tube and stored in fridge (45 min at −18 °C), and next, it was evaporated into dryness, and reconstitute in 1.2 mL acetonitrile. 

After starting the preparation of the sample (d-SPE step), the solid residue was discarded and the extract was transferred into a 15 mL polypropylene tube with single salts or mixtures (e.g., 30 mg Z-Sep and 50 mg primary secondary amine (PSA)). Next, the tube was shaken for 1 min, stored in a fridge (10 min), and centrifuged for 15 min (6000 rpm, 3480 rcf). The acetonitrile supernatant (~800 uL) was obtained with a pipette and evaporated to dryness. Mixture MeOH: water (20:80, *v/v*) was added to the dry residue and the extract was reconstitute in 200 uL and analyzed by HPLC-DAD andLC–QqQ-MS equipment. The flowchart of the procedure is presented in Figure 1. 

### 3.4. RP-HPLC

#### 3.4.1. HPLC-DAD

Agilent Technologies 1200 HPLC system with a quaternary pump was used for the LC analysis. Analytes were separated using a Scherzo SM-C18 150 mm × 4.6 mm column, with a 3-µm particle size (Imtakt, Portland, OR, USA). The column was thermostated at 22 °C. Mobile phase consisted of 50 mM HCOOH in water (component A) and 50 mM HCOOH in MeCN (component B). Gradient elution at 0.4 mL min^−1^ mobile phase flow was applied. The gradient program was as follows: 0–10 min from 40% eluent B to 100% B; 10–16 min isocratic 100% B. Final samples were injected onto the column using a Rheodyne manual injector with 20 µL loop. 

Detection was carried out simultaneously at four different wavelengths (220, 230, 240, 260, and 280 nm). Identification of bisphenols was accomplished on the basis of the retention times of the analytes and by comparison between the UV spectra of the detected peaks of the samples and UV spectra of the reference compounds in the chromatograph. 

Quantitative analysis in this regard was carried out on the basis of single-point calibration, in which response factors were calculated as amount-to-area ratios of the analytes in the calibration sample and used in the analyte-concentration calculation in fortified samples. 

#### 3.4.2. LC-QqQ-MS

Chromatographic analysis was performed using HPLC (Agilent 1260; Germany). The separation was carried out using a Poroshell 120 EC-C18 3.0 × 100 mm column with a 2.7 μm particle size (Agilent Technologies) at 40 °C. Two mobile phases were used during chromatographic experiments. After optimization of mobile phase, a mixture of water + 5 mM ammonium acetate (A), and acetonitrile-water (9 + 1, *v/v*) + 5 mM ammonium acetate (pH 7.9) (B) was used in gradient elution mode. The gradient program was as follows: 0–13 min from 20% eluent B to 95% B; 13–13.10 min from 95% eluent B to 100% B; 13.10–14 min isocratic 100%B. Furthermore, the mobile phase consisted of 50 mM ammonium buffer adjusted to pH = 4.00 by adding formic acid (component A) and 0.03% ammonia in methanol (component B) was applied in gradient elution mode. The gradient program was as follows: 0–1 min from 5% eluent B to 50% B; 1–4 min from 50% eluent B to 80% B; 4–7 min isocratic 80%B; 7–15 min from 80% eluent B to 50% B. Source parameters (ESI): ion spray voltage, 40 kV for ESI (+); collision gas temperature, 300 °C; collision gas flow, 10 L/min; nebulizer, 40 psi; sheath gas temperature, 400 °C; and sheath gas flow, 12 L/min. Source parameters were optimized as showed in Table 4 [18]. 

### 3.5. Method Optimization and Validation

#### 3.5.1. HPLC Method Validation

The standard calibration curves of the analytes were constructed by plotting analyte concentration against peak area. Bisphenols standards prepared as solutions in methanol were prepared at seven concentrations in the range from 250 to 2000 ng mL^−1^ for HPLC-DAD (Table 1) for systems in the unconjugated form (without incubation with β-glucuronidase) and conjugated form (with incubation with β-glucuronidase); and also 1 to 50 ng mL^−1^ LC-QqQ-MS (Table 2, for selected bisphenols such as BPA, BPS, and BPF), and injected in triplicate under the same chromatographic conditions. The calibration curves of bisphenols under investigation showed satisfactory linearity and correlation between concentration and peak area over the studied range with the determination coefficient, *R*^2^, ≥0.9881. The instrumental limits of quantification (LOQ) for all analytes were calculated using following formula (Equation (1)):

(1) $$LOQ=10\frac{SD}{S}$$

where *SD* is the standard deviation of y-intercept of regression lines (calculated using the LINES function in MS Excel 2010), and *S* is the slope of the calibration plot. Retention times and full calibration data including instrumental LOQs are presented in Table 1. 

#### 3.5.2. Recovery and Precision Studies

Breast milk samples were spiked with the bisphenols under investigation at following concentrations levels: 5, 50, and 500 ng mL^−1^ (5 and 50 ng mL^−1^ for LC-QqQ-MS; 500 ng mL^−1^ for HPLC-DAD). Samples were fortified with the appropriate volume of the working standard mixture. Recovery studies were performed on the basis of seven replicates from the spiking procedure (n = 7) at each concentration level. Relative standard deviations expressed as a percentage (% RSD) were calculated for all of the analytes. Method limits of quantification (mLOQs) were set as the minimum spiking level (ng mL^−1^) that can be quantified with acceptable accuracy and precision. 

Recovery studies for milk samples from a can, which were fortified at 500 ng mL^−1^, were in the range of 15-135% with RSD% ≤ 18% (Figure 3 and Figure 4). Recovery values for individual analytes were such as: for BPS (135%, RSD 18%), BPF (38%, RSD 12%), BPA (18% RSD 10%), and BPB (15% RSD 7%). Recovery values for BADGE and derivatives were equals 10% for BADGE * H_2_O or less (7% and 3% for BADGE * HCl and BADGE, respectively). 

The procedure was transferred for recovery studies bisphenols in breast milk samples. Recovery studies for breast milk samples, which were fortified at 500 ng mL^−1^, were in the range of 15-107% with RSD% ≤ 10% (Figure 5). Recovery values for individual analytes were following: for BPS (107%, RSD 10%), BPF (60%, RSD 8%), BPA (38% RSD 8%) and BPB (15% RSD 5%). Recovery values for BADGE and derivatives were no more than 3% (Figure 5). 

#### 3.5.3. Degree of the Matrix Interference Assessment

Influence of matrix interferences on the proper quantification of the analytes was assessed as a percentage difference in a signal of the bisphenols in final matrix compared to the signals obtained for standards in injection solvent. For this purpose, the approach similar to this proposed by Kruve et al. [21] was applied. Sets of samples was prepared in blank breast milk extracts (for each woman) reconstituted with known standard solution amounts (n = 3 for each concentration level), while the second sets consisted of standard solutions at the same concentrations. The concentration levels selected for this study correspond to the concentrations of the analytes in final extracts obtained from samples spiked at 5 and 50 ng mL^−1^, respectively, assuming 100% recovery for each analyte. Finally, degree of the matrix interference (MI%) was calculated on the basis of triplicates (n = 3) according to following equation (Equation (2)):

(2) $$MI\%=\left(\frac{A_{Post\;extraction\;spike}\;}{A_{Standard}}-1\right)\times 100\%$$

where *A* denotes mean peak areas of the standard (*A_Standard_*) and the breast matrix extract (*A_Post extraction spike_*) spiked at the same concentration level. Ideally, a value of 0 is related to the absence of matrix interference. Values of MI% calculated for all the analytes under investigation are presented (Table 2). 

## 4. Conclusions

The present study addresses the presence of selected bisphenols in milk samples from a can and breast milk samples. In this paper, analytical procedures for selected bisphenols residue testing in natural and biological samples with application QuEChERS-based extraction procedure and HPLC-DAD and LC–QqQ-MS. Described procedures allowed obtaining satisfactory recovery rates of the studied analytes. Proper extract purification was achieved using a mixture of 50 mg PSA and 30 mg Z-Sep (and also 150 mg MgSO_4_); the application has a pivotal role in lipids removal from initial extracts. 

Analysis of biological samples confirms feasibility of the developed QuEChERS-HPLC-DAD and QuEChERS-LC-QqQ-MS procedure in BPS, BPF, and BPA, determined by the breast milk samples. The frequency of detection of total BPA was more than BPS and BPF. To our knowledge, this is the largest biomonitoring study of bisphenols in breast milk samples in Poland. 

β-Glucuronidase allows the determination of the associated bisphenols (in the form of conjugated analytes). β-Glucuronidase has a positive effect on the sample preparation process (especially sweetened milk from a can) using the QuEChERS technique, by reducing the viscosity of the samples, which facilitates the process of proper mixing. 

This procedure can be successfully applied to the analysis of milk samples using various chromatographic techniques. Depending on the chromatographic technique used, at the end of the procedure the samples are dissolved in various solvents. For analysis by HPLC-DAD or LC-QqQ-MS, the analytes are concentrated in a mixture of methanol and water at the final extract. The sample at the end of procedure must be derivatized before being analyzed by HPLC-FLD and GC-MS/MS. Furthermore, in the case of GC-MS/MS analysis, the final extract should be dissolved/concentrated in non-polar or less polar solvents (e.g., hexane and tetrbutyl ether). 

In the present (breast milk samples (n = 75)) and near future (breast milk samples (n = 75)) those factors that could influence the bisphenols levels and estimated the exposure and the risk for breast fed infants will also be studied. 

## Figures and Tables

**Table 1:** Validation data including retention time, optimal wavelength, calibration curve and range, *R*^2^, and method limits of detection (LOD) and quantification (LOQ) for bisphenols in breast milk samples obtained after proposed QuEChERS-HPLC-DAD procedure.

No	Analyte	*t_R_* (min)	*λ* (nm)	Range (ng mL^−1^)	Without Incubation with β-Glucuronidase				Incubation with β-Glucuronidase			
					Calibration Curve	*R* ^2^	LOD (ng mL^−1^)	LOQ (ng mL^−1^)	Calibration Curve	*R* ^2^	LOD (ng mL^−1^)	LOQ (ng mL^−1^)
**1**	BPS	~7.3	280	250–2000	y = 0.0907x − 1.8053	0.9881	140	423	y = 0.0893x + 2.4926	0.9717	665	2014
**2**	BPF	~9.1	280	250–2000	y = 0.0505x − 0.179	0.9948	53	159	y = 0.0506x − 1.233	0.980	549	1663
**3**	BPA	~10.35	280	250–2000	y = 0.0482x − 4.1544	0.989	92	277	y = 0.043x + 5.1191	0.9689	142	430
**4**	BADGE *H_2_O	~10.8	280	250–2000	y = 0.0206x − 0.6918	0.9883	76	230	y = 0.0192x − 1.8587	0.9894	693	2102
**5**	BPB	~11.375	280	250–2000	y = 0.0392x + 0.0659	0.9952	237	720	y = 0.036x + 3.1957	0.957	165	500
**6**	BADGE * 2HCl	~13.75	280	250–2000	y = 0.0215x + 1.1725	0.9945	272	824	y = 0.0206x − 0.0784	0.987	366	1110
**7**	BADGE	~14.5	280	250–2000	y = 0.0245x − 1.4438	0.9894	306	926	y = 0.0143x + 1.9757	0.9657	260	787

**Table 2:** Validation data including calibration range, *R*^2^, SD of slopes and intercepts, recoveries (%), relative standard deviations expressed as a percentage (RSD%), degree of the matrix interference (MI%) for two levels such as 5 ng mL^−1^ and 50 ng mL^−1^, and method limits of quantification (mLOQ) for bisphenols in breast milk samples obtained after proposed QuEChERS-LC-QqQ-MS procedure.

Analyte	Calibration Data						Recovery ^c^ (%) (RSD%)	MI% ^d^	Recovery ^c^ (%) (RSD%)	MI% ^d^
	Range (ng mL^−1^)	Calibration Curve	*R* ^2^	SD of Slope ^a^	SD of Intercept ^a^	mLOQ ^b^ (ng mL^−1^)	5 ng mL^−1^	5 ng mL^−1^	50 ng mL^−1^	50 ng mL^−1^
**BPS**	1-50	y = 3986x + 7542	0.9999	68	950	0.25	102 (8)	12	95 (15)	13
**BPF**	1-50	y = 285x + 604	0.9998	13	180	0.13	68 (9)	11	63 (9)	10
**BPA**	1-50	y = 203x + 547	0.9991	11	150	0.10	39 (7)	10	35 (17)	12

**^a^** SD of slope and intercept were obtained using the LINEST function (MS Excel 2010), which returns an array of the statistics for a calculated trend line by using the least squares method. **^b^** mLOQ = method limit of quantification. Minimal residue concentration at which elaborated procedure enabled identification and quantification of the analyte with acceptable reliability and accuracy. **^c^** Average recoveries (and RSD%) of the analytes in breast milk samples (n = 7, at each spiking level). **^d^** MI% = mean degree of the matrix interference expressed as percentage difference in a signal from the analyte in matrix (final extract) compared to the signal in injection solvent. MI% was studied for analyte concentrations corresponding to 100% recovery at investigated fortification levels in triplicates (n = 3).

**Table 3:** BPA, BPS, BPF, and BPB residues identified in breast milk samples conducted from June to August in 2018, and from March to April 2019 from fifty healthy voluntary-women donors in Lublin (urban) and Lubelskie voivodeship (rural), Poland.

Breast Milk Sample	Concentration in Breast Milk Sampleng mL^−1^ (Urban Area)				Breast Milk Sample	Concentration in Breast Milk Sampleng mL^−1^ (Rural Area)			
	BPA	BPS	BPF	BPB		BPA	BPS	BPF	BPB
**1**	0.35	-	-	-	**26**	-	0.62	-	+
**2**	0.37	0.36	0.29	-	**27**	0.31	-	-	-
**3**	0.41	-	-	-	**28**	0.39	-	-	-
**4**	-	0.29	-	-	**29**	0.51	0.52	-	-
**5**	0.68	0.41	-	+	**30**	0.39	-	0.34	-
**6**	0.33	-	-	-	**31**	0.42	-	-	-
**7**	0.49	-	-	-	**32**	0.52	0.51	-	-
**8**	0.46	-	-	-	**33**	0.41	-	-	-
**9**	0.29	-	-	-	**34**	0.22	-	-	-
**10**	0.28	0.43	-	-	**35**	0.22	-	-	-
**11**	0.48	-	-	-	**36**	0.39	0.32	-	-
**12**	0.67	-	-	+	**37**	0.24	-	-	-
**13**	-	0.45	0.25	-	**38**	0.41	0.39	-	-
**14**	0.26	-	-	-	**39**	-	-	-	-
**15**	0.61	0.51	-	+	**40**	-	-	-	-
**16**	0.31	-	-	-	**41**	0.26	-	-	-
**17**	0.36	-	-	-	**42**	0.41	0.32	-	+
**18**	0.69	0.68	-	+	**43**	-	-	-	-
**19**	0.61	-	0.22	+	**44**	-	-	-	-
**20**	-	0.43	-	-	**45**	0.21	-	-	-
**21**	0.43	-	-	-	**46**	-	0.33	-	-
**22**	0.23	0.36	-	-	**47**	0.46	-	0.55	-
**23**	0.44	-	-	-	**48**	-	-	-	-
**24**	-	0.41	-		**49**	-	-	-	-
**25**	0.39	-	-	-	**50**	0.31	-	-	-

"+" means that BPB was identified in the sample (without its quantification).

**Table 4:** Detection was achieved using a QqQ 6460 spectrometer equipped with an electrospray ionization (ESI) Jet Stream source (Agilent Technologies). Source parameters were optimized as follows (ESI).

Compounds	Precursor Ion [m/z]	Product Ion [m/z]	Fragmentor [V]	Collision Energy [V]	Polarity
**d_16_-BPA**	241	225 * 151	100	15 30	negative negative
**d_8_-BPS**	249	257 * 112	166	20 20	negative negative
**d_10_-BPF**	209	199 * 97	98	20 20	negative negative
**BPA**	227	212 * 133	98	15 25	negative negative
**BPS**	249	108 * 156	166	20 25	negative negative
**BPF**	209	199 * 105	98	20 20	negative negative
**BADGE**	358	191 * 161	98	5 25	positive positive
**BADGE-2H_2_O**	394	209 * 135	98	20 30	positive positive
**BADGE-2HCl**	430	227 * 135	98	15 40	positive positive

* ion selected to quantitative analysis.
